# Carotenoids, Birdsong and Oxidative Status: Administration of Dietary Lutein Is Associated with an Increase in Song Rate and Circulating Antioxidants (Albumin and Cholesterol) and a Decrease in Oxidative Damage

**DOI:** 10.1371/journal.pone.0115899

**Published:** 2014-12-30

**Authors:** Stefania Casagrande, Rianne Pinxten, Erika Zaid, Marcel Eens

**Affiliations:** 1 Department of Biology, Ethology Group, University of Antwerp, Campus Drie Eiken, Antwerp, Belgium; 2 Institute for Education and Information Sciences, Research Unit Didactica, University of Antwerp, Antwerp, Belgium; Utrecht University, Netherlands

## Abstract

Despite the appealing hypothesis that carotenoid-based colouration signals oxidative status, evidence supporting the antioxidant function of these pigments is scarce. Recent studies have shown that lutein, the most common carotenoid used by birds, can enhance the expression of non-visual traits, such as birdsong. Nevertheless, the underlying physiological mechanisms remain unclear. In this study we hypothesized that male European starlings (*Sturnus vulgaris*) fed extra lutein increase their song rate as a consequence of an improved oxidative status. Although birdsong may be especially sensitive to the redox status, this has, to the best of our knowledge, never been tested. Together with the determination of circulating oxidative damage (ROMs, reactive oxygen metabolites), we quantified uric acid, albumin, total proteins, cholesterol, and testosterone, which are physiological parameters potentially sensitive to oxidation and/or related to both carotenoid functions and birdsong expression. We found that the birds fed extra lutein sang more frequently than control birds and showed an increase of albumin and cholesterol together with a decrease of oxidative damage. Moreover, we could show that song rate was associated with high levels of albumin and cholesterol and low levels of oxidative damage, independently from testosterone levels. Our study shows for the first time that song rate honestly signals the oxidative status of males and that dietary lutein is associated with the circulation of albumin and cholesterol in birds, providing a novel insight to the theoretical framework related to the honest signalling of carotenoid-based traits.

## Introduction

During the last decades, indicator traits signalling individual condition have attracted the attention of evolutionary ecologists with an increasing interest in the physiological constraints limiting the expression of characters honestly signalling (*sensu* Zahavi [1]) genetic or phenotypic quality [2]. A principal physiological hypothesis holds that an indicator trait can convey information about the capacity of an individual to maintain a high functionality of the biochemical transformations for which it is crucial to control the production of reactive oxygen species (ROS). ROS are common by-products of the aerobic metabolism, and central components of the somatic state of the organism [3], but an over-production of ROS can lead to a suboptimal production of energy due to a negative effect of oxidative damage on mitochondrial and cell functionality [2], [3]. In accordance with this, a signalling trait, such as birdsong, could inform us about the efficiency of the redox pathway [3], [4]. For example, it has been recently shown that a genetic deficiency in enzymatic antioxidants impairs the olfactory sexual signalling in mice [5].

The song in passerine birds can be seen as a cognitive expression that involves several brain functions such as perception, learning, memory and synchronization of well-defined neural nuclei [6]. Cognitive performances can be potentially sensitive to an increase of ROS because the brain possesses a relative low concentration of antioxidants, high concentrations of oxidation-sensitive lipids and several enzymes producing ROS [7]. Both repertoire size and song rate are potentially affected by the redox state. However, repertoire size is generally more related to the conditions experienced during the diverse developmental phases of the individual, while song rate may better mirror present conditions [8], [9]. Therefore, it is generally plausible to foresee that a lower cell efficiency related to an imbalance of ROS can constrain song expression.

Another side of the relationship between vocal signals and oxidative state is that the production of song itself could alter the redox state, as a protracted singing performance can represent a metabolic cost. This because singing is a demanding activity associated with the costly context-dependent development of song-control nuclei in the brain [10], [11] and an increase in the energetic expenditure [12] that can double the metabolic rate of calling birds [13], with the consequent raise of ROS. Nevertheless, evidence of the existence of clear metabolic costs of birdsong is still mixed [14], [15].

In some instances, an overproduction of ROS can occur also in other circumstances, such as during increased locomotor activity [16], diverse reproductive phases [17] or during the activation of the immune system [18]. This can result in a condition of oxidative stress when the organism does not possess adequate defences to neutralize ROS [19]. Animals rely on both endogenous (intra-cell enzymes and non-enzymatic molecules) and dietary antioxidants (vitamin E and A, flavonoids, polyphenols and carotenoids) to minimize the non-targeting action of reactive species [20] on cell membranes and vital biomolecules [19]. Among dietary antioxidants, carotenoids, which are pigments absorbed through the diet by animals and traded-off between signalling and health maintenance functions [21]–[23], have been extensively studied. Indeed, it is known that they are effective in quenching ROS, at least *in vitro* [24], and are part of the antioxidant barriers of animals, at least in some cases [25]–[27]. However, in birds, one of the most studied taxonomical classes in this field of research [28], convincing evidence indicating that carotenoids are antioxidants remains elusive [29], [30].

This mixed evidence could be explained by the fact that carotenoids are not directly used as antioxidants, but they can promote the biosynthesis of more powerful antioxidants. Supporting this hypothesis is the evidence that they can activate the transcription system of antioxidant enzymes such as NAD(P)-quinineoxido-reductase, γ-glutamylcysteinsynthetase, in addition to other proteins with antioxidant functions [31], [32]. The capacity of carotenoids to control the biosynthesis of molecules has been largely neglected in ecological studies, while it could represent the key element to understand their physiological role, not just in relation to their direct antioxidant potential. Indeed, they can induce lipogenic processes [33] and activate the biosynthesis of sex steroids [34].

The above evidence indicates that carotenoids are involved in several vital processes that are independent to the production of visual sexual signalling. In line with this, a recent study revealed that male European starlings (*Sturnus vulgaris*) provided with lutein increase their song rate [35]. This observation is notable due to the fact that while it is known that song rate is related to the nutritional state of the performing individual [36], the underlying mechanism as to why a specific calorie-free micronutrient such as lutein affects song, remains unknown. Possibly, since song rate is a condition-dependent cognitive expression [37], [38] and since the brain is particularly vulnerable to oxidative stress [4], lutein may have ameliorated the oxidative status by promoting the antioxidant network. While von Schantz et al. [4] in their seminal review have already argued that birdsong is especially sensitive to an imbalance of ROS (see also [3]), this has, to the best of our knowledge, not yet been tested.

To address this possibility, we experimentally studied the relationship between dietary lutein, song rate and physiological condition to investigate if song performance boosted by dietary lutein can honestly signal the health condition of male European starlings during winter. In this period, males are not reproductively active and their beak does not yet have the typical yellow lutein-dependent colouration [39]. Male starlings exhibit a high song rate throughout the year without a significant difference being observed between the reproductive and non-reproductive season for both intensity and patterns of performance [40], [41]. This unique feature enabled us to study singing males outside the breeding season, while investigating the function of carotenoids in a signalling context without taking into account their role as pigments. Moreover, at this time we can control for any anti- or pro-oxidant effect of circulating sex steroids [42], [43], which are at their minimum during this part of the year. Previous studies indicated that male starling song outside of the breeding season serves to establish and/or maintain the dominance in hierarchies as well as acting in defence of nest holes [40], [44]. Furthermore, song rate is related to individual quality [36] and shows a within-individual consistency between the non-reproductive and reproductive season [41]. Additionally, it is known that song bout length is related to the cognitive performance in this species [45] and is a trait selected by females during mating [46], [47].

We investigated through which physiological pathway dietary lutein can exert its boosting effect on song rate, assessing: 1) the antioxidant function of lutein [27] by quantifying specific components of the antioxidant plasmatic barrier together with circulating markers for lipid, nucleic acid and protein peroxidation (reactive oxygen metabolites, ROMs); 2) the steroidogenic action of lutein [34] by quantifying testosterone, which is related to the metabolism of carotenoids and cholesterol [48], can affect song rate [41], [49] and can act as pro- or anti-oxidant [42], [43]; 3) the lipogenic action of lutein [33] by measuring cholesterol, which can influence the availability of lutein [48] and is positively associated to the song rate of birds [36]; 4) the proteogenic effect of lutein [31], [32] by assessing circulating albumin and total proteins since albumin is positively associated with song rate [36] and both albumin and total proteins can have antioxidant properties [50].

Specifically, we predict that: 1) song rate honestly signals the oxidative status of the organism (being positively related to antioxidants and negatively to oxidative damage); and, 2) lutein administration increases song rate and that this effect is associated with the up-regulation of circulating antioxidants. Although there is some evidence that signalling traits can honestly mirror the redox state [51]–[53], our study is the first to investigate this relationship in the context of birdsong.

## Materials and Methods

### Ethics statement

The capture of wild European starlings *Sturnus vulgaris* and their housing in captivity for scientific purpose were approved by the Agency for Nature and Forest (ANB) of the Flemish government (permit number: 08-11344). This study was performed in Antwerp (GPS coordinates: N 51° 13′ 11.9532″, E 4° 23′ 46.4532″) and conformed to the Belgian and Flemish legislation and was approved by the ethical committee for animal experiments (ECD) of the University of Antwerp (ID number: 2011-32). The European starling is not an endangered or protected species and has been shown to easily adapt to captivity and to show normal social behaviour in these conditions [35].

### Housing conditions

This study used 56 adult European starling males that were captured as juveniles in the Antwerp region in 2010 and kept in large outdoor aviaries at the campus of the University of Antwerp. All males were kept under similar captive conditions until the experiment started. One month prior to the experiment they were randomly assigned to 4 identical neighbouring outdoor aviaries, where they were then exposed to a natural decreasing photoperiod (from 09 h 22 min to 08 h 04min). The aviaries (L×W×H; 27.0×7.0×2.75 m), were each equipped with 14 nestboxes. Each nestbox was provided with a singing perch and other perches were located at several places in the aviary as described in Van Hout et al. [35]. Food and water were provided *ad libitum*. All starlings were marked with a unique combination of coloured bands, which allowed individual recognition from remote observation blinds. From the 6^th^ of November 2012 (day 0) until the 6^th^ of December (day 30), 28 birds received food enriched with lutein (Caro-group) while the other 28 males were kept under a standard maintenance diet (C-group). Each group was divided into two groups of 14 birds each in order to have 2 aviaries of equal size for both groups. Birds were randomly assigned to an aviary and group. Aviaries hosting Caro- and C-groups alternated with each other and birds of each cage were in visual and acoustic contact with birds of other cages.

### Lutein supplementation

Since we were interested in determining which was the physiological condition that could have determined the increase in the song behaviour already observed in the European starling [35], the dietary lutein supplementation was performed in accordance to the previous study. Briefly, we mixed 50 g of ORO GLO (Kemin Industries Inc., Iowa, USA; extracted from marigolds *Tagetes erecta* and containing 15.0 g*kg^−1^ of lutein) per 1 kg of the starlings’ standard food (mixture of 1/3 Orlux UniPatee, Orlux, Belgium and 2/3 Merelkorrel Speciaal, Nifra - Van Camp, Belgium). In both this and in the previous study we ascertained that this dose is effective in increasing the concentration of circulating lutein to the levels observed in free ranging males of the closely related spotless starling *Sturnus unicolor* during the reproductive season (9.20±0.64 µg*mL^−1^; [54]). Since plasma carotenoids usually fluctuate over time, with a maximum peak during the reproductive season and a minimum peak during the non-reproductive season [55], working in winter time allowed us to obtain two groups (high and low circulating carotenoids), both having circulating lutein within a natural range.

### Behavioural observations

On day 0 (before the start of the diet manipulation), day 15 and day 30 we blood sampled and measured the body mass of all birds. During the four days preceding each of the three sampling points we daily monitored simultaneously the song rate of all the starlings housed in one aviary, using a point sampling technique, with an interval of one minute. Each aviary was observed during the 4 consecutive days (one observation session of 45–60 min per day per aviary) between 09h00 and 13h00 alternating the order of the aviaries between subsequent days in order to have a balanced distribution of the timing of observation. Overall song rate was defined as the proportion of samples during which a male was singing (in any and all locations) compared to the total number of samples. Since nestbox-oriented song is more linked to the reproductive state and more regulated by the endocrine status than overall song rate [35], we also determined the proportion of the time spent performing nestbox-oriented song rate, which only includes song production on the perch attached to the nestbox or very close to or on the nestbox [49].

### Physiological parameters

We assessed several physiological components that could be regulated by lutein and that could be linked to birdsong. Specifically, since carotenoids can promote the synthesis of proteins we quantified circulating albumin, an important representative of thiols, like glutathione, which are organic compounds characterized by having a sulfhydryl group (-SH) with antioxidant properties [56]. Albumin is generally associated with birdsong performance [36] and total plasmatic proteins, indicators of condition, (e.g. [57] with a potential role in the antioxidant barrier [50]). We described the plasmatic antioxidant barrier also by quantifying uric acid, a by-product of protein catabolism strongly correlated with Total Antioxidant Capacity in birds (TAC, [58], [59]).

We determined circulating cholesterol because it can increase the availability of carotenoids in birds [48]. Although the inverse relationship, i.e. the effect of carotenoids on cholesterol, has never been investigated, we wanted to ascertain in detail the relationship between lutein and cholesterol for the following reasons: (1) it has been shown that carotenoids can promote lipogenesis [33]; (2) it could have a role in defending the organism from oxidation [60], [61] and (3) it is a precursor of sex steroids [62]. Thus we investigated the possibility that its variation in the peripheral blood could affect the hormonal status (testosterone) of singing birds. Indeed, it is known that, although starlings can sing also when castrated [63], testosterone can regulate song behaviour in this species [41], [49] and be related to the oxidative status as pro-oxidant [42] or anti-oxidant [43]. Moreover, since carotenoids can upregulate the production of pro-steroids we wanted to control for their potential effect on testosterone.

We also quantified the oxidative damage detectable in the blood to have a detailed picture of the oxidative status of individuals [64], to control for any potential pro-oxidant effect of carotenoids [29] and to examine if song rate honestly signals the oxidative status of birds.

### Blood sampling and quantification of circulating lutein

The physiological parameters were assessed in the peripheral plasma of birds immediately after capturing. Approximately 500 µL of blood was collected with a heparinised microvette by puncturing the brachial vein and kept in a cooling bag until centrifugation. Blood was centrifuged at 1500 g for 10 min within 4 hours from sampling and kept at -80°C until analysis (4 months later). Plasma lutein was extracted by vortexing 10 µL of plasma diluted 1∶10 in ethanol and centrifuging it for 10 min at 1500×g. The optical density of the supernatant was determined at 445 nm with a plate reader (VersaMax, Molecular Devices Inc, California, USA). Lutein concentration was determined from a standard curve of lutein (Cat. N. X6250, Sigma-Aldrich Co. LLC, Missouri, USA). Samples were analysed singularly. The mean intra plate CV of standards was 4.8% and the inter-plate CV was 5.5%. We run three plates and repeated samplings from the same individual were always analyzed within the same plate.

### Quantification of the non-enzymatic antioxidants barrier

We described the antioxidant defence by quantifying the levels of uric acid, albumin and total proteins circulating in the blood. These molecules have all been proven to have strong antioxidant activity (see [58] for uric acid; [56] for albumin; [50] for other proteins) and they are the main target of the most common protocols used to assess total antioxidant defence. Uric acid, for instance, is strongly associated with the antioxidants measured by quantifying the ferric reducing ability of plasma – FRAP assay [59] and it is also associated with trolox equivalent antioxidant capacity – TEAC/TAS/TAC assay or uric acid equivalents measured in AOP-490 assay [2], [58], [59]. Similarly, almost all proteins are the main target of the protocols that assess the concentration of plasmatic antioxidants quantifying the reaction of hypoclorous acid HOCl (e.g. OXY assay) [57], [65], a potent oxidant, which is also naturally produced by the organism during inflammation. We assessed also circulating cholesterol as a precursor of steroid hormones, promoter of circulating carotenoids and potential antioxidant (see introduction). Uric acid, albumin, total proteins (thiolic and non-thiolic proteins corrected for albumin levels by subtracting albumin values) and cholesterol were determined in 20 µL of plasma (diluted 1∶5 with distilled water) using an automatic chemistry analyser (ABX Pentra 400, Horiba Ltd., Kyoto, Japan). Repeatability (within-run precision) and reproducibility (run-to-run precision) of the measurements are certified by including a calibrator and a control (Horiba) with known concentrations each run [66], [67].

### Oxidative damage

The oxidative damage was quantified by measuring the levels of reactive oxygen metabolites (ROMs) with the d-ROM test (Cat. N. MC003, Diacron International srl, Grosseto, Italy). The ROMs detected by the test are hydroperoxides, which are products of lipid, protein and nucleic acid peroxidation. ROMs might also indicate potential future damage because hydroperoxides can be broken by Fenton reaction, generating free radicals. The assay was performed on 10 µL of plasma and 200 µL of the reactive solution provided with the kit. The absorbance was read with a microplate reader (VersaMax, Molecular Devices Inc, California, USA) at a wavelength of 505 nm in endpoint mode. Measurements are expressed as mmol*L^–1^ of H_2_O_2_ equivalents. All samples, standards and controls for high and low concentrations were run in duplicate. The mean intra-plate coefficient of variation (CV) of samples was 3.72%, while the inter-plate CV calculated from the standards was 1.2%.

### Endocrine status

Testosterone concentrations were determined using enzyme immunoassay (EIA) kits (Cat. No. ADI-901-065, Enzo Life Sciences, New York, USA) following a diethyl ether extraction of 25 µL sample volume. After drying the extract under N_2_ stream, 250 µL of Assay Buffer was added (1∶10 dilution), and the samples were allowed to reconstitute overnight at 4°C. A stripped plasma sample spiked with a known amount of testosterone (2 ng*mL-1) as well as one blank sample containing only assay buffer were taken through the entire assay procedure. The next day, 100 µL of each sample (in duplicate) was added to individual wells on the assay plate alongside a standard curve with 5 points ranging from 7.81 pg*mL-1 to 2,000.00 pg*mL-1. The samples were added randomly within and across plates but an individual’s repeated samples were always included on the same plate. The plate was read on microplate reader (VersaMax, Molecular Devices Inc, California, USA) at 405 nm with a correction wavelength set at 570 nm. The average extraction efficiency was of 65% and final values were corrected accordingly. The lower sensitivity of the assay was at 5.84 pg*ml-1 and the detection limit determined by the lower standard was 0.038 pg*ml-1). The mean intra-plate CV was 3.9%, while the inter-plate CV were calculated for three different concentrations of the standard curve and were, respectively, 6.6% (2,000 pg*ml-1), 1.1% (124 pg*ml-1) and 4.7% (7.8 pg*ml-1). Although individuals were bled within an average of 12.83±0.6 min (range: 1–33 min) from capture, testosterone levels were not related to the timing of bleeding (F_(1,154.07)_ = 0.12, p = 0.73).

### Data analysis

We tested the effect of lutein supplementation on respectively, overall song rate and nestbox-oriented song rate, by using a full factorial general linear mixed model (SAS 9.3 with SAS Enterprise Guide 5.1, SAS Institute Inc. NC). Treatment and time were specified as fixed factors and cage and individual nested into cage as random factors. Significant differences between and within groups were ascertained by pairwise differences of least square means. After checking the normality of residuals, only lutein and testosterone were log-transformed to reach normality. To assess which physiological mechanisms affected song behaviour we ran a generalized linear model for both song rates corresponding to day 30, together with all the physiological variables measured on day 30 as covariates after checking for collinearity (all Variance Inflation Factors, VIF<2.04). Best models were ranked among all the possible combination using the Akaike’s Information Criterion (AIC) for model building and by calculating ΔAIC for each model compared to the best one. Among the 127 combinations of models only the ones with Δ<1 were reported [68]. Model building analyses were made with Statistica (version 10.0, StatSoft, Inc. Tulsa, USA).

## Results

### Behavioural responses

#### Overall song rate

**Figure 1:**
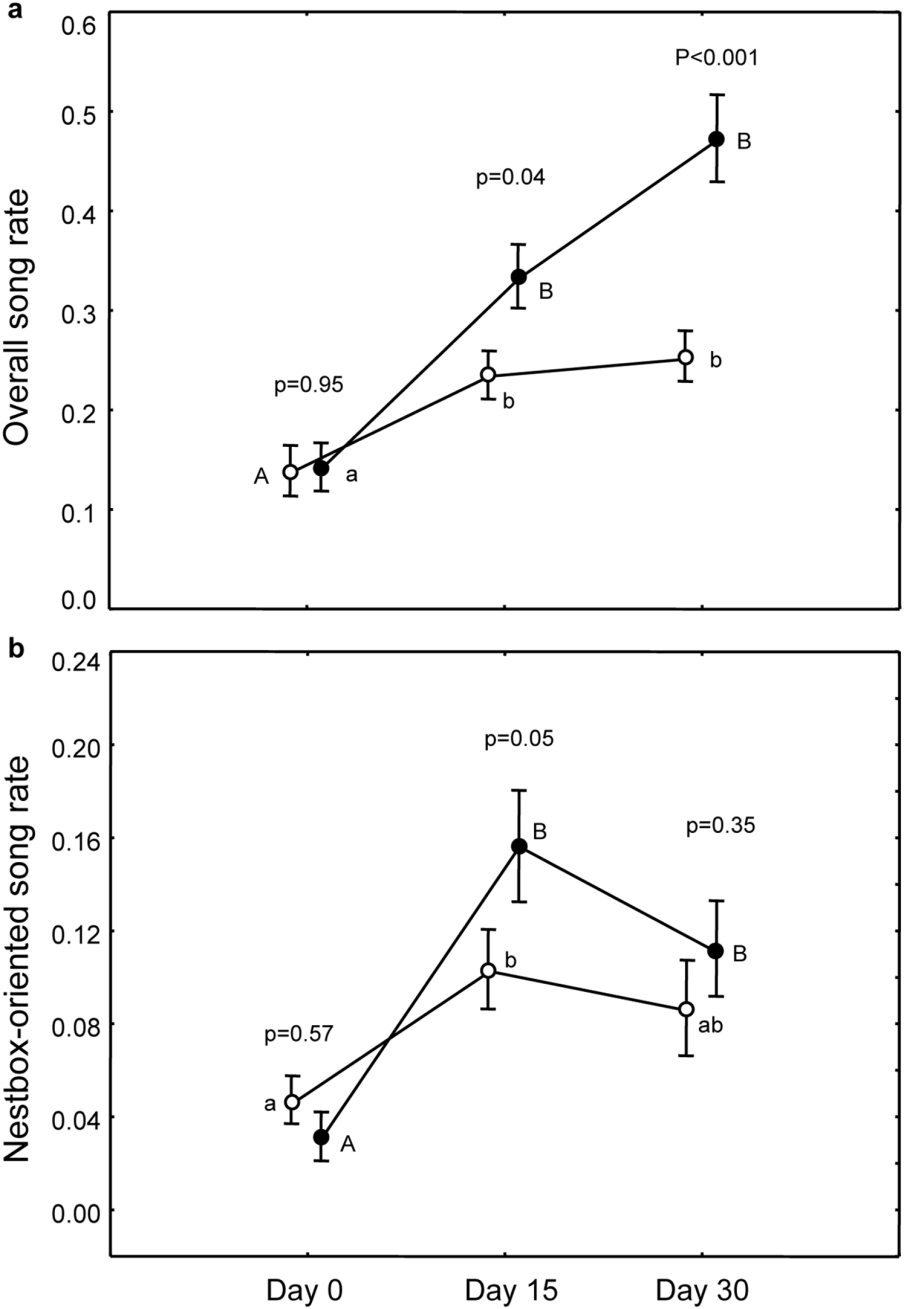
Effect of dietary carotenoids on song rate. Variation during time of overall song rate (a) and nestbox-oriented song rate in the two groups (full circles for Caro-birds). P values refer to pairwise differences of least square means between groups within the same day (reported only when significant) and within each group between different days (different letters indicate significant difference with song rate observed on day 0; capital for Caro-group and small letters for C-group).

The two groups did not differ in overall song rate before treatment (p = 0.95). Birds treated with lutein sang more than controls both at day 15 and day 30 (treatment×time, F_(2,108)_ = 12.60, p<0.0001; treatment F_(1,108)_ = 7.21, p = 0.008; time, F_(2,108)_ = 53.94, p<0.0001; between groups post-hoc p values shown in Fig. 1a). Both groups significantly increased their song rate with time (Caro_0_–Caro_15_: p<0.0001; Caro_0_–Caro_30_: p<0.0001; C_0_–C_15_: p = 0.002; C_0_–C_30_: p = 0.0003; Fig. 1a).

#### Nestbox-oriented song rate

The two groups sang at the same rate before treatment (p = 0.57) increasing song rate over time (time, F_(2,108)_  = 19.66, p<0.0001) with a nearly significant treatment×time interaction (F_(2,108)_ = 2.79, p = 0.07; treatment, F_(1,108)_  = 1.06, p = 0.31, Fig. 1b). Post-hoc analyses showed that nestbox-oriented song rate of birds treated with lutein was higher than controls on day 15 (Fig. 1b) and that the increase of nestbox-oriented song was more pronounced in treated birds (Caro_0_–Caro_15_: p<0.0001; Caro_0_–Caro_30_: p<0.0002) than in controls (C_0_–C_15_: p = 0.008; C_0_–C_30_: p = 0.06; Fig. 1b).

### Body mass

On day 0 the two groups did not differ in body mass (p = 0.44). Body mass increased during time irrespectively of treatment (overall means observed on day 0: 80.09±0.61 g, day 15: 82.16±0.54 g, day 30: 83.38±0.69 g; time, F_(2,108)_ = 21.63, p<0.0001; treatment×time, F_(2,108)_ = 0.12, p = 0.88; treatment, F_(1,108)_ = 0.34, p = 0.91).

### Physiological responses

#### Lutein

**Figure 2:**
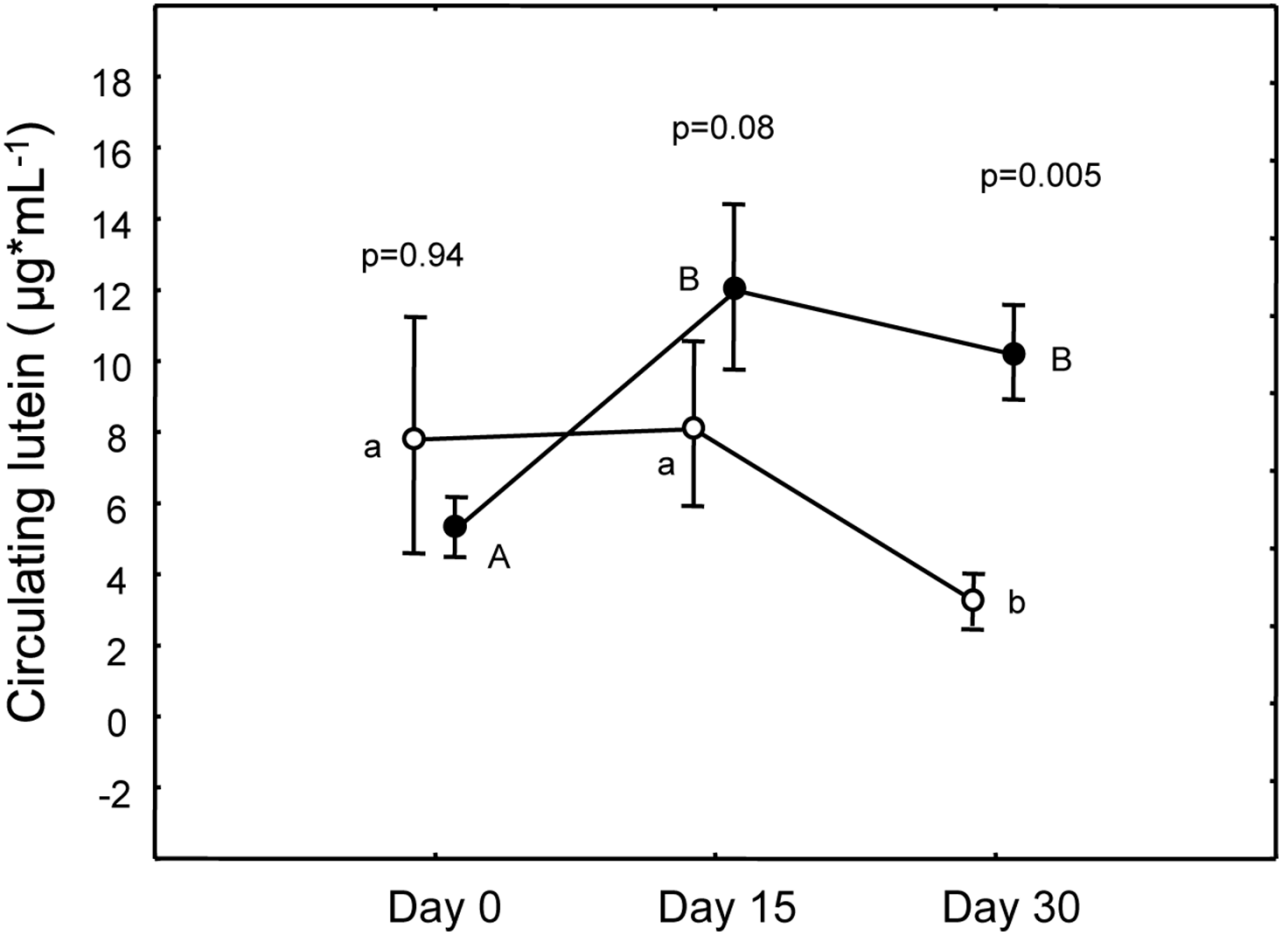
Variation of circulating carotenoids in the two treatments. Full circles represent Caro-birds. P values refer to pairwise differences of least square means between groups within the same day (reported only when significant) and within each group between different days (different letters indicate significant difference with what observed on day 0; capital for Caro-group and small letters for C-group).

Groups did not differ in circulating lutein before the experiment (p = 0.94, Fig. 2). Treated birds increased circulating lutein already from day 15, while controls decreased it (treatment×time, F_(2,108)_ = 9.62, p = 0.0001; treatment, F_(1,108)_ = 2.78, p = 0.10; time, F_(2,108)_ = 4.03, p = 0.02, Fig. 2, post-hoc: Caro_0_–Caro_15_: p = 0.0002; Caro_0_–Caro_30_: p = 0.0004; C_0_–C_15_: p = 0.98; C_0_–C_30_: p = 0.02; Fig. 2). Compared to C-males, Caro-males had nearly significantly higher lutein values on day 15 and highly significantly higher values on day 30 (Fig. 2).

#### Albumin

**Figure 3:**
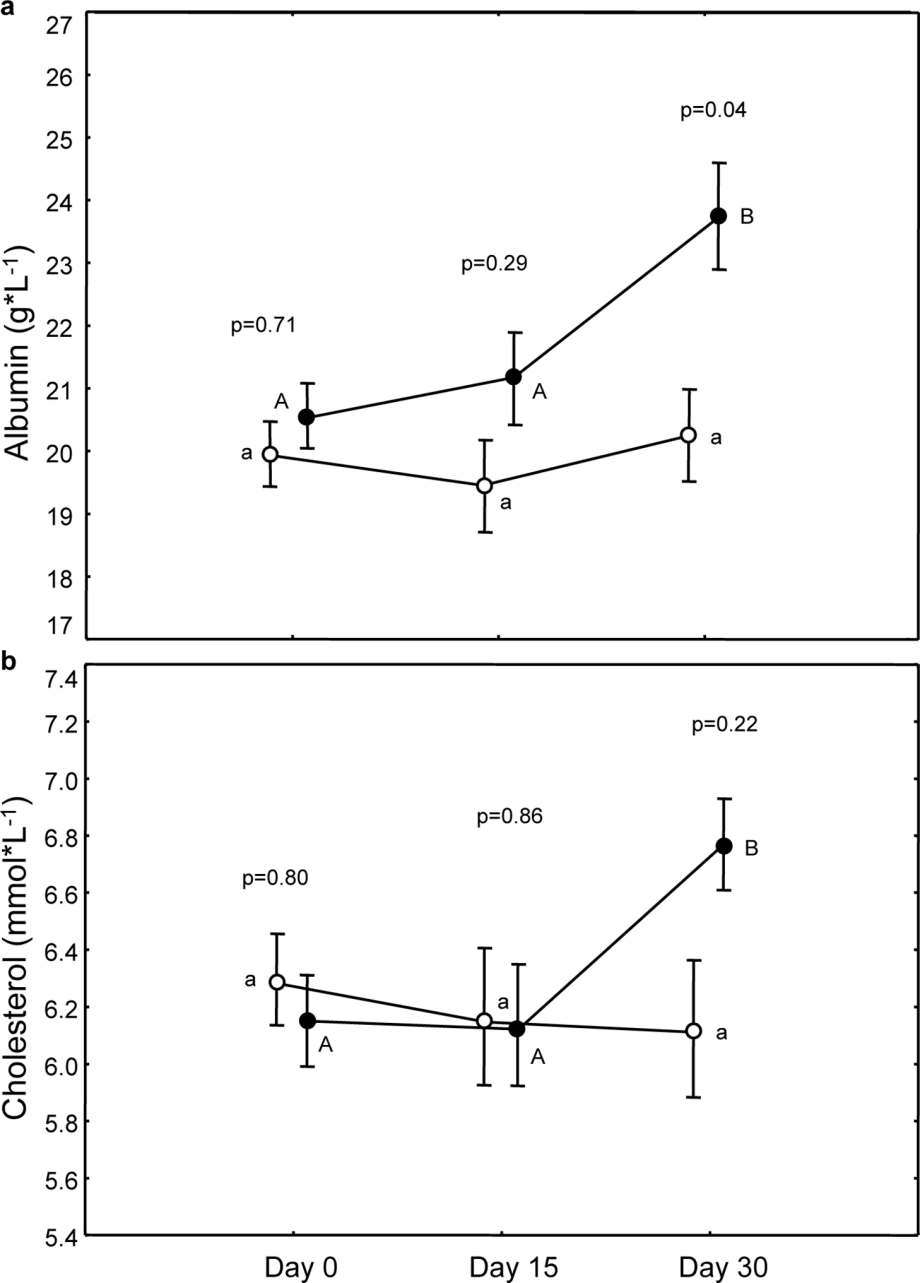
Variation of albumin and cholesterol during time in the two treatments. Full circles represent Caro-birds. P values refer to pairwise differences of least square means between groups within the same day (reported only when significant) and within each group between different days (different letters indicate significant difference with what observed on day 0; capital for Caro-group and small letters for C-group).

Before the experiment the two groups showed similar levels of circulating albumin (p = 0.71). Birds fed lutein significantly increased albumin with time while controls did not (treatment×time, F_(2,108)_ = 3.68, p = 0.03; treatment, F_(1,108)_ = 1.66, p = 0.20; time, F_(2,108)_ = 6.86, p = 0.002). The increase of circulating albumin was significant only on day 30 (post-hoc: Caro_0_–Caro_15_: p = 0.40; Caro_0_–Caro_30;_ p = 0.001 C_0_–C_15_: p = 0.54; C_0_–C_30_: p = 0.67; Fig. 3a). On day 30 Caro-males had significantly higher levels of circulating albumin than C-males (p = 0.04) while on 15 days after the start of the treatment the levels of albumin between the two groups were similar (C_15_-Caro_15_: p = 0.29; Fig. 3a).

#### Cholesterol

Groups did not differ in circulating cholesterol before the treatment (p = 0.80). Only Caro-birds showed an increase in cholesterol levels during time (treatment×time, F_(2,108)_ = 4.43, p = 0.01; treatment, F_(1,108)_ = 0.10, p = 0.75; time, F_(2,108)_ = 2.39, p = 0.10; post-hoc: Caro_0_–Caro_15_: p = 0.90; Caro_0_–Caro_30_: p = 0.003; C_0_–C_15_: p = 0.50; C_0_–C_30_: p = 0.40; Fig. 3b).

#### Uric acid

Uric acid did not differ between the groups on day 0 (p = 0.39) and it did not vary with any of the factors considered (treatment×time, F_(2,108)_ = 0.06, p = 0.94; treatment, F_(1,108)_ = 1.70, p = 0.19; time, F_(2,108)_ = 0.50, p = 0.61; overall means: controls, 700.30±38.12 µmol*L^−1^; carotenoids 785.83±39.38 µmol*L^−1^).

#### Total proteins

Total proteins did not differ between the groups on day 0 (p = 0.33) and increased over time in both treatments (time, F_(2,108)_ = 24.57, p<0.0001; treatment×time, F_(2,108)_ = 1.86, p = 0.16; treatment, F_(1,108)_ = 1.42, p = 0.24; overall means: day 0: 15.64±0.77 g*L^−1^; day 15: 15.72±0.89 g*L^−1^; day 30: 19.55±1.04 g*L^−1^).

#### Oxidative damage

**Figure 4:**
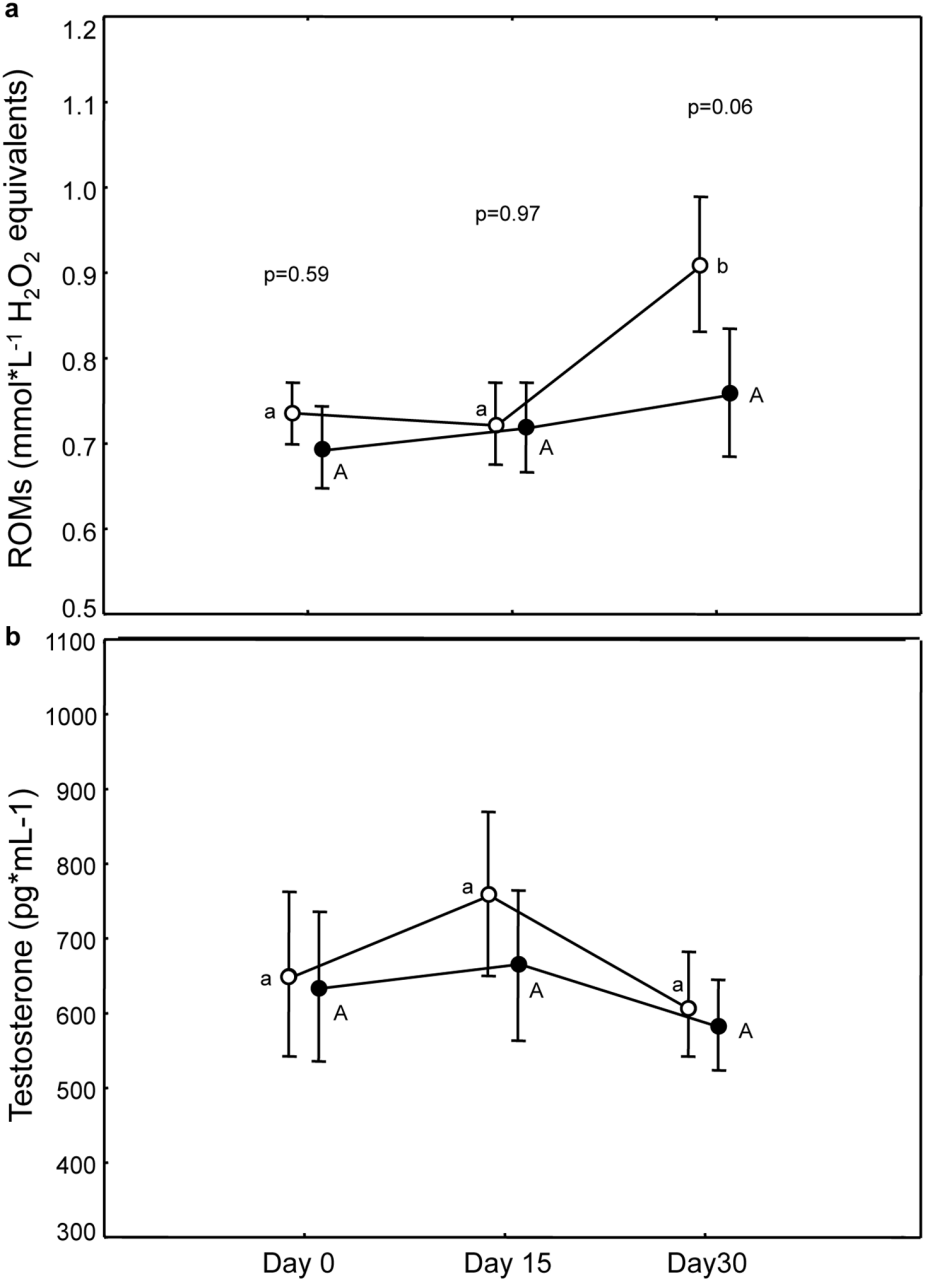
Variation of oxidative damage (a) and testosterone (b) in the two treatments. P values refer to pairwise differences of least square means between groups within the same day. Post-hoc results within each group and between different days results are represented by letters (different letters indicate significant difference with what observed on day 0; capital for Caro-group and small letters for C-group).

ROMs were similar in the two groups before the treatment (p = 0.59) and increased during time (time, F_(2,108)_ = 5.31, p = 0.006). Although we did not find a significant interaction between treatment and time (F_(2,108)_ = 1.77, p = 0.18; treatment, F_(1,108)_ = 0.99, p = 0.32; Fig. 4a) this increase was evident only for C-birds after 30 days (post-hoc: C_0_–C_30_: p = 0.004, all other p>0.26 both for C- and Caro-birds; a nearly significant difference between C- and Caro-birds was registered on day 30: p = 0.06).

#### Testosterone

Circulating testosterone was similar in the two groups before the treatment (p = 0.68) and it did not vary with time, treatment or their interaction (treatment×time, F_(2,108)_ = 0.29, p = 0.75; treatment, F_(1,108)_ = 0.01, p = 0.96; time, F_(2,108)_ = 0.88, p = 0.42, Fig. 4b).

### Relationship between song rate and physiological variables

**Figure 5:**
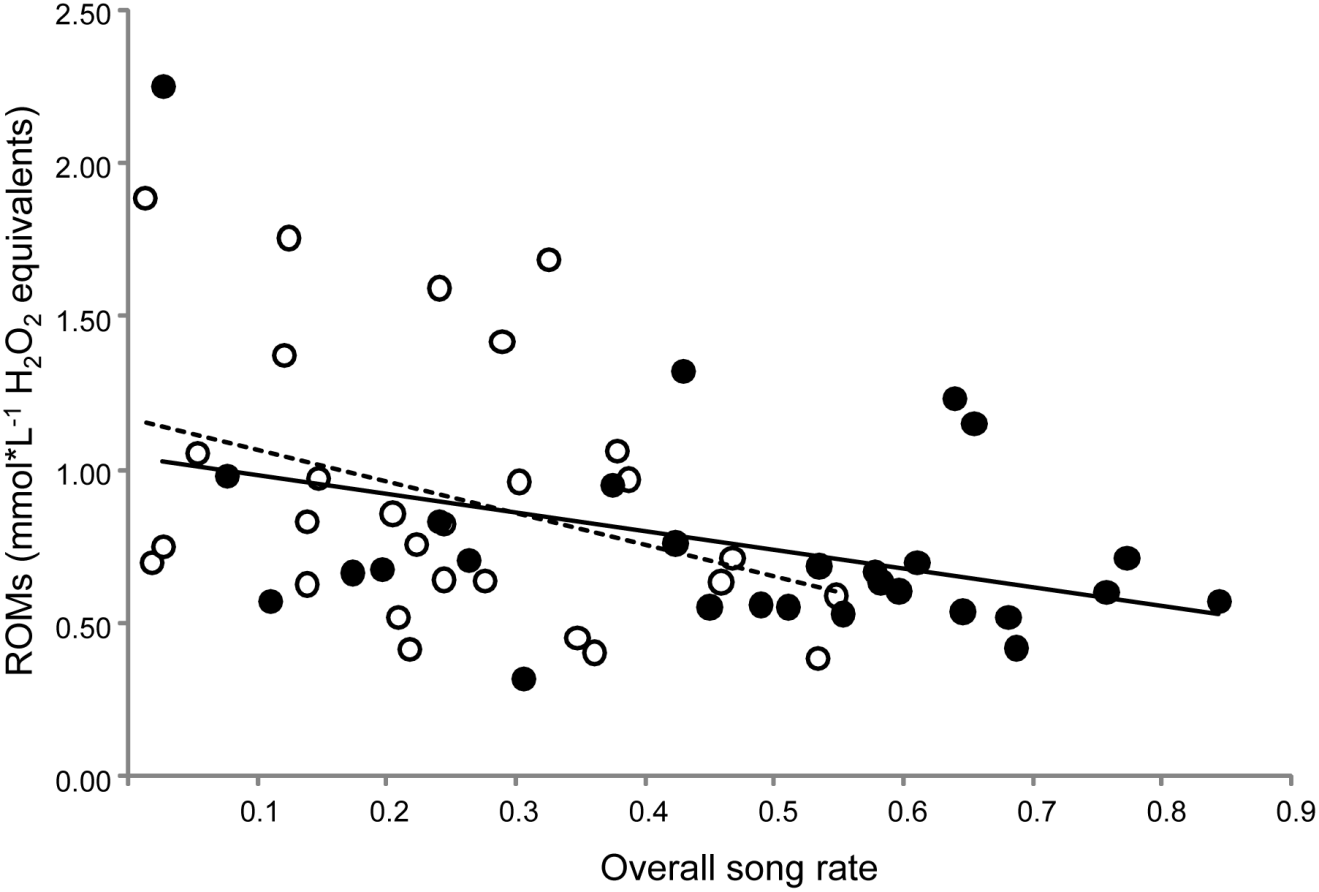
Relationship between overall song rate and oxidative damage in the two treatments on day 30. Full dot/solid trendline for Caro-group and open dots/dashed trendline for C-birds.

The models built to understand which physiological variables explained the variation in song rate observed at day 30 showed that both overall song rate and nestbox-oriented song rate were positively affected by circulating levels of cholesterol and albumin while being suppressed by the oxidative damage (ROMs) and total proteins (Tables 1,2; Fig. 5). Overall song rate was also predicted by lutein while nestbox-oriented song rate was predicted also by circulating testosterone (Tables 1,2).

**Table 1:** Models related to overall song rate (a) and nestbox-oriented song rate (b) and all the physiological variables measured on day 30.

*a. Overall song*	*ΔAIC*	*L ratio*	*p*
Chol/ROMs/Caro/TP	0.00	22.46	0.001
Alb/Chol/ROMs/Caro/TP	0.80	23.66	0.001

*b. Nestbox song*	*ΔAIC*	*L ratio*	*P*
Chol/TP	0.00	9.27	0.01
Alb/Chol/TP/T	0.59	10.12	0.02
Chol/TP/ROMs	0.90	9.94	0.02

Note: Chol: cholesterol; TP: total proteins; Alb: albumin; Caro: circulating lutein; ROMs: reactive oxygen species; UA: uric acid; T: testosterone; ΔAIC: difference in Akaike information criterion between each model with the best model; L ratio: likelihood ratio test.

**Table 2:** Estimates related to the models of Table 1 describing the association between overall song rate (a) and nestbox-oriented song rate (b) with the physiological variables measured on day 30.

a. Overall song rate	
*Variable*	*Estimate±se*
** Alb**	**0.03±0.02**
** Chol**	**0.12±0.06**
** TP**	**−0.02±0.01**
UA	0.0±0.0
** Caro**	**0.41±0.16**
** ROMs**	**−0.65±0.26**
T	0.0±0.0

b. Nestbox-oriented song rate	
*Variable*	*Estimate±se*
** Alb**	**0.04±0.05**
** Chol**	**0.30±0.18**
** TP**	**−0.09±0.03**
UA	0.0±0.0
Caro	0.16±0.27
** ROMs**	**−0.39±0.22**
** T**	**0.78±0.63**

Variables retained in models explaining song rate variation (reported in Table 1) are indicated in bold.

Note Abbreviations are specified in Table 1.

## Discussion

In accordance with our first prediction, both overall and nestbox-oriented song rates were positively associated with circulating antioxidants - albumin and cholesterol - and negatively with oxidative damage, clearly showing that they honestly reflect the redox status of singing males. In addition, we show for the first time, at least in animals other than humans, that birds receiving extra lutein showed an increase of circulating albumin and cholesterol, two abundant and effective plasmatic antioxidants, as foreseen by our second prediction. This suggests that, at least when carotenoids have not to be traded-off between health maintaining and signalling functions, they are key elements of the expression of a non-visual secondary sexual trait. Our study confirmed that male starlings provided with extra lutein in their diet during the non-breeding season sing more than controls [35], but we now show that this effect is not mediated by the variation in circulating testosterone levels or in body mass. Lastly, we did not find any effect of the treatment on uric acid or total proteins.

We will discuss these points firstly explaining the significance of an up-regulation of albumin and cholesterol when considering the redox status, then addressing in the last part of the discussion the relevant results on the relationship between singing behaviour and oxidative status.

### The role of albumin and cholesterol

Birds treated with lutein showed an increase in circulating albumin and cholesterol. This effect can probably be ascribed to the role of carotenoids in controlling the biosynthesis of proteins and lipids (see Introduction), although at least three other alternative explanations could be advanced to explain the direct effect of lutein on albumin and cholesterol. Lutein could have reduced the clearance of albumin and cholesterol by e.g. promoting their accumulation via antioxidant protection, or, they could have facilitated their mobilization from diverse tissues. Another explanation is that the increase of song activity caused the up-regulation of these antioxidants. Further studies should clarify if the action of dietary lutein on albumin and cholesterol is direct or indirect.

Besides the biochemical pathway, we think that our findings are relevant because both albumin and cholesterol are potent antioxidants. It has been estimated that in human blood approximately 70% of free radicals can become trapped due to circulating antioxidants like albumin [56]. Albumin is a protein produced in the liver that contains one cysteine residue, which constitutes the largest reduced pool of thiols in the circulation [56], [69], [70]. Due to its chemical properties, albumin is able to scavenge both radicals and non-radicals oxidants [71]. It can bind reactive ions such as copper and iron, which are very potent in generating hydroxyl radicals after reacting with oxygen or hydrogen peroxide. Another antioxidant activity of albumin comes from its ability to transport bilirubin, specifically inhibiting lipid peroxidation [71]. Although albumin is known to have such a strong antioxidant role [72] it has never been considered as such by evolutionary ecologists, despite the fact that it has been measured in several studies involving birds as a general indicator of health status or nutrition (e.g. [73], [36]).

Besides its role as antioxidant, albumin is also involved in regulating several physiological processes as it is a non-specific carrier of carotenoids, hormones and lipids [56]. Among the latter, the ability to bind oxysterols is particularly important as cholesterol can be oxidized forming oxysterols (see further discussion below). All of this evidence supports the view that the increase of circulating lutein resulted in the increase of a potent thiol, boosting the plasmatic antioxidant barrier.

We also found that birds fed with lutein upregulated the circulation of cholesterol. This up-regulation should be interpreted as a promotion of the antioxidant network as well. The antioxidant role of cholesterol appears to be related to its synthesis as one of the enzymes involved (3β-hydroxysterol-Δ24-reductase) has to use a hydrogen peroxide to produce cholesterol. In general, the biosynthesis of cholesterol is an oxygen-intensive process that could have enabled the adaptation of tetrapods to the terrestrial life [61]. Its ‘bad’ reputation is related to the fact that it is transported in the watery blood mainly by low-density lipoproteins (LDL, also called “bad cholesterol” although they are not with synonymous of cholesterol), which are very sensitive to oxidation and produce the atherosclerosis plaque when oxidized. Although cholesterol produces active oxysterols when oxidized, these metabolites are rapidly sequestrated by carriers such as albumin [56] and degraded in the liver. By means of this fast degradation, their concentration is kept at very low levels [60]. In our study we found an increase in the level of albumin that could have minimized the presence of oxysterols. In line with the beneficial function of cholesterol as an antioxidant, a recent study carried out on the rainbow trout (*Oncorhynchus mykiss*), indicated that dietary cholesterol increased blood and hepatic activity of both enzymatic (superoxide dismutase, glutathione-peroxidase and catalase) and non-enzymatic antioxidants, decreasing the oxidative damage assessed by measuring hepatic malondialdehyde (MDA, [74]). The relation we found between song rate, antioxidants and oxidative damage (also explained below) suggests that the role of albumin and cholesterol, usually associated with the nutritional condition, should not be interpreted merely as a regulation of the trade-off of resources in favour of the production of an energetically demanding exhibition of a costly signal, such as singing.

### Song rate and redox status

The upregulation of two antioxidants, albumin and cholesterol, observed in birds treated with lutein was associated with an increased expression of song rate. Together with the fact that both overall song rate and nestbox-oriented song rate were inversely related to the levels of oxidative damage and positively related to plasmatic antioxidants, we can conclude that both measures of song rate signalled the redox status of the singing male. This is in agreement with several recent studies that reported an association between oxidative status and secondary sexual traits, but to the best of our knowledge our study is the first to report this for birdsong. For example, it has been found that less carotenoid-based coloured great tit males *Parus major* suffer a greater reduction in sperm motility, swimming ability, and increased levels of sperm lipid peroxidation compared to more colourful males [51] and that yellowthroat males *Geothlypis trichas* with brighter yellow bibs produced by carotenoids showed lower levels of DNA damage [52]. Although Von Schantz et al. [4] already mentioned song as the more plausible trait among sexual ornaments to be sensitive to oxidative stress, we are not aware of any studies that have addressed this possibility to date. Song behaviour can be particularly sensitive to oxidative stress because it is a cognitive expression of the brain, an organ that consumes oxygen at a high rate, has low concentrations of antioxidants, is abundant in oxidation-sensitive lipids and that possesses several enzymes producing ROS [7]. Thus, it is plausible that the oxidative status can be a limiting factor in the expression of song, because uncontrolled levels of ROS can constrain the mitochondrial respiration, and thus the correct functioning of the physiological processes underpinning song behaviour [3], [75].

Our findings that 1) C-birds sang less than Caro-birds but showed increasing ROMs levels during time while this was not the case for Caro-birds; 2) song rate and oxidative damage were negatively associated with each other; and 3) the variation in ROMs levels explained both overall song and nestbox-oriented song, all suggest that the song rate of starling males was constrained by the oxidative damage of the organism, and that Caro-birds who controlled the increase of ROMs, possibly by increasing circulating levels of albumin and cholesterol, performed better. This is in line with the hypothesis that the expression of sexual ornaments reflects the exposure to oxidative stress and the competence of fighting ROS [4]. Although Caro- and C-birds clearly showed a different pattern of ROMs variation during time, we did not find a statistically significant effect of dietary lutein on ROMs levels. This is not surprising since we found that the effect of lutein on oxidative damage is probably not direct (lutein did not act as antioxidant itself; [29]), but rather indirect, as lutein acted as a promoter of other antioxidants (see Introduction). From a signalling perspective this explanation would be an extension of the view advanced by Hartley & Kennedy [29]. They argued that carotenoids would signal the ability to cope with oxidative damage not because of their antioxidant function but because of their ability to promote and signal the expression of other potent antioxidants. Further research should investigate this novel role of lutein during the reproductive phase and considering visual traits as well.

Our study cannot indicate the origin of the observed oxidative damage and further studies are needed to clarify this aspect. We can exclude that birdsong itself was the cause of an increased ROMs level because the association between the two was negative. This result indirectly provides support to studies that did not find a metabolic cost of birdsong performance [13], [14]. We could speculate that the positive trend of ROMs observed with time could be associated to a natural seasonal trend observed also in other species, such as in the Eurasian kestrel *Falco tinnunculus* [43].

We used the supplement ORO GLO to increase lutein in our experimental birds, as was done in many previous studies where lutein was provided with the food [76]–[85]. This product contains a low percentage of lutein. The other known ingredients of this supplement are grain by-products, amorphous silicon dioxide and ethoxiquin. The latter is an artificial antioxidant added to preserve the pigments from oxidation. We did not control for these additional ingredients but we think that it is highly unlikely that our results were attributable to or affected by them for the following reasons: 1. the standard food provided to starlings already represented a control for grain by-products, since its main composition is represented by corn and wheat together with their by-products. Considering the low amount of OroGlo added to the total amount of food provided we do not think that it represented a significant extra source of calories derived from grains; 2. the amorphous silicon dioxide is a calorie-free food additive commonly used in animal nutrition, which is also hardly absorbed by the gut; 3. the effect of ethoxiquin can be excluded because it has been shown that it can decrease circulating cholesterol [86] and can cause proteinuria (high concentration of proteins in urine and a resultant low concentration in the blood) [87]. We can exclude both conditions because we measured increased levels of cholesterol (already reported in the literature for other model species) and increased levels of albumin.

### Song rate and testosterone

Until now, most studies trying to explain variation in the expression of song among males have focused on hormones [88] and on the neural substrate [89]. With regard to the hormonal regulation of song, our study confirms previous results obtained in European starlings [49], [66]: while overall song rate was not related to T at day 30, part of the variation in nestbox-oriented song rate was explained by T.

### Ecological implications

Our study has important ecological implications as it is one of the first to show that ‘food quality’ affects the expression of an acoustic trait in songbirds. A striking result is, indeed, that oxidative state was significantly improved in birds eating lutein-rich food. Thus, a high-quality diet allowed birds to control the oxidative damage that can constrain song performance, and at the end, to sing at a higher rate. Therefore, the physiological condition of the bearer was mediated, in this case, by the environmental condition represented by the quality of food, indicating that birdsong is a complex signal conveying specific information about the physiological state of the individual [3], [4], and to a wider extent, about the quality of the territory and/or of the foraging quality of the male.

Future studies should address the functional role of the interplay between oxidative status and lutein in affecting the expression of different song traits, both outside and during the reproductive period, and in conjunction with the allocation of these pigments to the production of carotenoid-based beak colouration during the reproductive period. Oxidative stress biomarkers were measured in the blood only, and they might not entirely reflect the oxidative conditions of specific regions of the brain involved in the production of song, such as the HVC nucleus. It would be valuable to measure oxidative stress in brain samples in future studies, and relate this to measures of song performance.

## References

[1] Zahavi A (1975) Mate selection-a selection for a handicap. J Theor Biol 53:205–14. 1195756 10.1016/0022-5193(75)90111-3

[2] Garratt M, Brooks RC (2012) Oxidative stress and condition-dependent sexual signals: more than just seeing red. Proc R Soc B 279:3121–3130 10.1098/rspb.2012.0568) PMC3385731 22648155

[3] Hill GE (2011) Condition-dependent traits as signals of the functionality of vital cellular processes. Ecol Lett 14:625–634 10.1111/j.1461-0248.2011.01622.x) 21518211

[4] von Schantz T, Bensch S, Grahn M, Hasselquist D, Wittzell H (1999) Good genes, oxidative stress and condition-dependent sexual signals. Proc R Soc B 266:1–12 10.1098/rspb.1999.0597) PMC1689644 10081154

[5] Garratt M, Pichaud N, Glaros EN, Kee AJ, Brooks RC (2014) Superoxide dismutase deficiency impairs olfactory sexual signaling and alters bioenergetic function in mice. Proc Natl Acad Sci U S A 111:8119–8124 10.1073/pnas.1322282111) 24843175 PMC4050573

[6] Buchanan KL, Grindstaff JL, Pravosudov VV (2013) Condition-dependence, developmental plasticity, and cognition: implications for ecology and evolution. Trends Ecol Evol 28:290–296 10.1016/j.tree.2013.02.004) 23518414 PMC3640828

[7] Sorce S, Krause KH (2009) NOX enzymes in the central nervous system: from signaling to disease. Antioxid Redox Signaling 11:2481–2504 10.1089/ARS.2009.2578) 19309263

[8] Ritschard M, Laucht S, Dale J, Brumm H (2011) Enhanced testosterone levels affect singing motivation but not song structure and amplitude in Bengalese finches. Physiol Behav 102:30–5 10.1016/j.physbeh.2010.10.005) 20951153

[9] Kubli SP, MacDougall-Shackleton E (2014) Developmental timing of signals affects information content: song complexity but not consistency reflects innate immune strategy in male song sparrows. Am Nat 183:660–70 10.1086/675757) 24739198

[10] Riters LV, Eens M, Pinxten R, Ball GF (2002) Seasonal changes in the densities of α_2_-noradrenergic receptors are inversely related to changes in testosterone and the volumes of song control nuclei in male European starlings. J Comp Neurol 444:63–74 10.1002/cne.10131) 11835182

[11] MacDonald IF, Kempster B, Zanette L, MacDougall-Shackleton S (2006) Early nutritional stress impairs development of a song-control brain region in both male and female juvenile song sparrows (*Melospiza melodia*) at the onset of song learning. Proc R Soc B 273:2559–2564 10.1098/rspb.2006.3547) PMC1634898 16959649

[12] Hasselquist D, Bensch S (2008) Daily energy expenditure of singing great reed warblers *Acrocephalus arundinaceus* . J Avian Biol 39:384–388 10.1111/j.2008.0908-8857.04427.x)

[13] Ophir G, Schrader SB, Gillooly JF (2010) Energetic cost of calling: general constraints and species-specific differences. J Evol Biol 23:1564–1569 10.1111/j.1420-9101.2010.02005.x) 20456573

[14] Ward S (2004) Singing is not energetically demanding for pied flycatchers, *Ficedula hypoleuca* . Behav Ecol 15:477–484 10.1093/beheco/arh038)

[15] Zollinger SA, Goller F, Brumm H (2011) Metabolic and respiratory costs of increasing song amplitude in zebra finches. PLoS ONE 6:e23198 10.1371/journal.pone.0023198) 21915258 PMC3168434

[16] Metcalfe NB, Alonso-Alvarez C (2010) Oxidative stress as a life-history constraint: the role of reactive oxygen species in shaping phenotypes from conception to death. Funct Ecol 24:984–996 10.1111/j.1365-2435.2010.01750.x)

[17] Stier A, Reichert S, Massemin S, Bize P, Criscuolo F (2012) Constraint and cost of oxidative stress on reproduction: correlative evidence in laboratory mice and review of the literature. Front Zool 9:37 10.1186/1742-9994-9-37) 23268929 PMC3551645

[18] Costantini D, Møller AP (2009) Does immune response cause oxidative stress in birds? A meta-analysis. Comp Biochem Physiol A 153:339–344 10.1016/j.cbpa.2009.03.010) 19303455

[19] Halliwell BH, Gutteridge JMC (2007) Free radicals in biology and medicine. 4th edn. Oxford University Press, Oxford. 851 p.

[20] Seifried HE, Anderson DE, Fisher EI, Milner J (2007) A review of the interaction among dietary antioxidants and reactive oxygen species. J Nutr Biochem 18:567–579 10.1098/rspb.1999.0597) 17360173

[21] Blount JD (2004) Carotenoids and life-history evolution in animals. Archives Biochem Biophys 430:10–15 10.1016/j.abb.2004.03.039) 15325906

[22] Vinkler M, Albrecht T (2010) Carotenoid maintenance handicap and the physiology of carotenoid-based signalisation of health. Die Naturwissenschaften 97:19–28 10.1007/s00114-009-0595-9) 19680618

[23] Svensson PA, Wong BBM (2011) Carotenoid-based signals in behavioural ecology: a review. Behaviour 148:131–189 10.1163/000579510X548673)

[24] Mortensen A, Skibsted LH, Truscott TG (2001) The interaction of dietary carotenoids with radical species. Arch Biochem Biophys 385:13–19 10.1006/abbi.2000.2172) 11361009

[25] El-Agamey A, Lowe GM, McGarvey DJ, Mortensen A, Phillip DM, et al (2004) Carotenoid radical chemistry and antioxidant/pro-oxidant properties. Archives Biochem Biophys 430:37–48 10.1016/j.abb.2004.03.007) 15325910

[26] Ewen JG, Thorogood R, Karadas F, Pappas AC, Surai PF (2006) Influences of carotenoid supplementation on the integrated antioxidant system of a free living endangered passerine, the hihi (*Notiomystis cincta*). Comp Biochem Physiol A 143:149–154 10.1016/j.cbpa.2005.11.006) 16406271

[27] Pérez-Rodríguez L (2009) Carotenoids in evolutionary ecology: re-evaluating the antioxidant role. BioEssays 31:1116–1126 10.1002/bies.200900070) 19705366

[28] Costantini D (2008) Oxidative stress in ecology and evolution: lessons from avian studies. Ecol Lett 11:1238–1251 10.1111/j.1461-0248.2008.01246.x) 18803642

[29] Hartley RC, Kennedy MW (2004) Are carotenoids a red herring in sexual display? Trends Ecol Evol 19:353–354 10.1016/j.tree.2004.04.002) 16701285

[30] Costantini D, Møller AP (2008) Carotenoids are minor antioxidants for birds. Funct Ecol 22:367–370 10.1111/j.1365-2435.2007.01366.x)

[31] Zhang L, Cooney RV, Bertram JS (1992) Carotenoids up-regulate connexin43 gene expression independent of their provitamin A or antioxidant properties. Cancer Res 52:5707–5712. 1327514

[32] Ben-Dor A, Steiner M, Gheber L, Danilenko M, Dubi N, et al (2005) Carotenoids activate the antioxidant response element transcription system. Mol Cancer Ther 4:177–186. 15657364

[33] Sahin K, Onderci M, Sahin N, Gursu MF, Khachik F, et al (2006) Effects of lycopene supplementation on antioxidant status, oxidative stress, performance and carcass characteristics in heat-stressed Japanese quail. J Therm Biol 31:307–312 10.1016/j.jtherbio.2005.12.006)

[34] Kazuyoshi T, Takeshi Y (1995) Avian neurosteroids. I. Pregnenolone biosynthesis in the quail brain. Brain Res 678:1–9 10.1016/0006-8993(95)001168) 7620878

[35] Van Hout AJM, Eens M, Pinxten R (2011) Carotenoid supplementation positively affects the expression of a non-visual sexual signal. PLoS ONE 6:e16326 10.1371/journal.pone.0016326) 21283591 PMC3026812

[36] Van Hout AJM, Pinxten R, Geens A, Eens M (2012a) Non-breeding song rate reflects nutritional condition rather than body condition. PLoS ONE 7:e36547 10.1371/journal.pone.0036547) 22590563 PMC3348915

[37] Boogert NJ, Giraldeau LA, Lefebvre L (2008) Song complexity correlates with learning ability in zebra finch males. Anim Behav 76:1735–1741 10.1016/j.anbehav.2008.08.009)

[38] Sewall KB, Soha JA, Peters S, Nowicki S (2013) Potential trade-off between vocal ornamentation and spatial ability in a songbird. Biol Lett 9:16–18 10.1098/rsbl.2013.0344) PMC3730647 23697642

[39] Müller W, Eens M (2009) Elevated yolk androgen levels and the expression of multiple sexually selected male characters. Horm Behav 55:175–181 10.1016/j.yhbeh.2008.09.012) 18976657

[40] Eens M (1997) Understanding the complex song of the European starling: An integrated ethological approach. Adv Stud Behav 26:355–434 10.1016/s0065-3454(08)60384-8)

[41] Van Hout AJM, Eens M, Balthazart J, Pinxten R (2009) Complex modulation of singing behavior by testosterone in an open-ended learner, the European Starling. Horm Behav 56:564–573 10.1016/j.yhbeh.2009.09.010) 19800345

[42] Alonso-Alvarez C, Bertrand S, Faivre B, Chastel O, Sorci G (2007) Testosterone and oxidative stress: the oxidation handicap hypothesis. Proc R Soc B 274:819–825 10.1098/rspb.2006.3764) PMC2093982 17251089

[43] Casagrande S, Costantini D, Dell’Omo G, Tagliavini J, Groothuis TGG (2012) Differential effects of testosterone metabolites oestradiol and dihydrotestosterone on oxidative stress and carotenoid-dependent colour expression in a bird. Behav Ecol Sociobiol 66:1319–1331 10.1007/s00265-012-1387-3)

[44] Ball GF, Hulse SH (1998) Birdsong. Am Psychologist 53:37–58 10.1037/0003-066X.53.1.37) 9442582

[45] Farrell TM, Weaver K, An YS, MacDougall-Shackleton SA (2011) Song bout length is indicative of spatial learning in European starlings. Behav Ecol 23:101–111 10.1093/beheco/arr162)

[46] Eens M, Pinxten R, Verheyen RF (1991) Male song as a cue for mate choice in the European starling. Behaviour 116:210–238.

[47] Gentner T, Hulse S (2000) Female European starling preference and choice for variation in conspecific male song. Anim Behav 59:443–458 doi:10.1006/anbe.1999.1313. 10675267

[48] McGraw KJ, Parker RS (2006) A novel lipoprotein-mediated mechanism controlling sexual attractiveness in a colorful songbird. Physiol Behav 87:103–108 10.1016/j.physbeh.2005.09.001) 16202433

[49] Pinxten R, De Ridder E, Balthazart J, Eens M (2002) Context-dependent effects of castration and testosterone treatment on song in male European starlings. Horm Behav 42:307–318 10.1006/hbeh.2002.1824) 12460590

[50] Medina-Navarro R, Durán-Reyes G, Díaz-Flores M, Vilar-Rojas C (2010) Protein antioxidant response to the stress and the relationship between molecular structure and antioxidant function. PLoS ONE 5:e8971 10.1371/journal.pone.0008971) 20126468 PMC2813298

[51] Helfenstein F, Losdat S, Møller AP, Blount JD, Richner H (2010) Sperm of colourful males are better protected against oxidative stress. Ecol Lett 13:213–222 10.1111/j.1461-0248.2009.01419.x) 20059524

[52] Freeman-Gallant CR, Amidon J, Berdy B, Wein S, Taff CC, et al (2011) Oxidative damage to DNA related to survivorship and carotenoid-based sexual ornamentation in the common yellowthroat. Biol Lett 7:429–432 10.1098/rsbl.2010.1186) 21247942 PMC3097884

[53] Moreno J, Velando A, Ruiz-de-Castañeda R, González-Braojos S, Cantarero A (2012) Oxidative damage in relation to a female plumage badge: evidence for signalling costs. Acta Ethol 16:65–75 10.1007/s10211-012-0138-9)

[54] Navarro C, Pérez-Contreras T, Avilés JM, Mcgraw KJ, Soler JJ (2010) Beak colour reflects circulating carotenoid and vitamin A levels in spotless starlings (*Sturnus unicolor*). Behav Ecol Sociobiol 64:1057–1067 10.1007/s00265-010-0920-5)

[55] Casagrande S, Dell’omo G, Costantini D, Tagliavini J, Groothuis T (2011) Variation of a carotenoid-based trait in relation to oxidative stress and endocrine status during the breeding season in the Eurasian kestrel: a multi-factorial study. Comp Biochem Physiol A Mol Integr Physiol 160:16–26 doi:10.1016/j.cbpa.2011.04.011. 21620990

[56] Roche M, Rondeau P, Singh NR, Tarnus E, Bourdon E (2008) The antioxidant properties of serum albumin. FEBS Lett 582:1783–1787 10.1016/j.febslet.2008.04.057) 18474236

[57] Meitern R, Sild E, Lind MA, Männiste M, Sepp T, et al (2013) Effects of endotoxin and psychological stress on redox physiology, immunity and feather corticosterone in greenfinches. PLoS ONE 8:e67545 10.1371/journal.pone.0067545) 23805316 PMC3689720

[58] Cohen A, Klasing K, Ricklefs R (2007) Measuring circulating antioxidants in wild birds. Comp Biochem Physiol B 147:110–21 10.1016/j.cbpb.2006.12.015) 17303461

[59] Costantini D (2011) On the measurement of circulating antioxidant capacity and the nightmare of uric acid. Methods Ecol Evol 3:321–325 10.1111/j.2041-210X.2010.00080.x)

[60] Smith LL (1991) Another cholesterol hypothesis: cholesterol as antioxidant. Free Radical Biol Med 11:47–61. 1937129 10.1016/0891-5849(91)90187-8

[61] Brown AJ, Galea AM (2010) Cholesterol as an evolutionary response to living with oxygen. Evolution 64:2179–2183 10.1111/j.1558-5646.2010.01011.x) 20394667

[62] Harvey R, Ferrier DR (2011) Biochemistry. Lippincott Williams & Wilkins, Baltimore. pp. 219–244.

[63] Pinxten R, De Ridder E, De Cock M, Eens M (2003) Castration does not decrease non-reproductive aggression in yearling male European starlings (*Sturnus vulgaris*). Horm Behav 43:394–401 10.1016/S0018-506X(03)00012-6) 12695113

[64] Costantini D, Verhulst S (2009) Does high antioxidant capacity indicate low oxidative stress? Funct Ecol 23:506–509 10.1111/j.1365-2435.2009.01546.x)

[65] Pattison DI, Hawkins CL, Davies MJ (2009) What are the plasma targets of the oxidant hypochlorous acid? A kinetic modeling approach. Chem Res Toxicol 22:807–817 10.1021/tx800372d) 19326902

[66] Geens A, Dauwe T, Eens M (2009) Does anthropogenic metal pollution affect carotenoid colouration, antioxidative capacity and physiological condition of great tits (*Parus major*)? Comp Biochem Physiol C 150:155–63 10.1016/j.cbpc.2009.04.007) 19394439

[67] Van Hout AJM, Pinxten R, Darras VM, Eens M (2012) Testosterone increases repertoire size in an open-ended learner: An experimental study using adult male European starlings (*Sturnus vulgaris*). Horm Behav 62:563–568 10.1016/j.yhbeh.2012.09.008) 23036784

[68] Burnham KP, Anderson DR (2002) Model selection and multi-model inference. Springer-Verlag, New York.

[69] Taverna M, Marie AL, Mira JP, Guidet B (2013) Specific antioxidant properties of human serum albumin. Ann Intensive Care 3:4–11 10.1186/2110-58203-4) 23414610 PMC3577569

[70] Turell L, Radi R, Alvarez B (2013) The thiol pool in human plasma: The central contribution of albumin to redox processes. Free Radical Biol Med 65:244–253 10.1016/j.freeradbiomed.2013.05.050) 23747983 PMC3909715

[71] Stocker R, Glazer N, Ames BN (1987) Antioxidant activity of albumin-bound bilirubin. Proc Natl Acad Sci 84:5918–5922. 3475708 10.1073/pnas.84.16.5918 PMC298974

[72] Hõrak P, Cohen A (2010) How to measure oxidative stress in an ecological context: methodological and statistical issues. Funct Ecol 24:960–970 10.1111/j.1365-2435.2010.01755.x)

[73] Tummeleht L, Mägi M, Kilgas P, Mänd R, Hõrak P (2006) Antioxidant protection and plasma carotenoids of incubating great tits (*Parus major* L.) in relation to health state and breeding conditions. Comp Biochem Physiol C 144:166–172 10.1016/j.cbpc.2006.08.004) 17035099

[74] Deng J, Kang B, Tao L, Rong H, Zhang X (2013) Effects of dietary cholesterol on antioxidant capacity, non-specific immune response, and resistance to *Aeromonas hydrophila* in rainbow trout (*Oncorhynchus mykiss*) fed soybean meal-based diets. Fish Shellfish Immunol 34:324–331 10.1016/j.fsi.2012.11.008) 23207478

[75] Hill GE, Johnson JD (2013) The mitonuclear compatibility hypothesis of sexual selection. Proc R Soc B 280:20131314 10.1098/rspb.2013.1314) PMC3757968 23945683

[76] Fitze PS, Tschirren B, Gasparini J, Richner H (2007) Carotenoid-based plumage colors and immune function: is there a trade-off for rare carotenoids? Am Nat 169:S137–44 10.1086/510094) 19426088

[77] McGraw KJ, Ardia DR (2003) Carotenoids, immunocompetence, and the information content of sexual colors: an experimental test. Am Nat 162:704–12 10.1086/378904) 14737708

[78] Bertrand S, Faivre B, Sorci G (2006) Do carotenoid-based sexual straits signal the availability of non-pigmentary antioxidant? J Exp Biol 209:4414–4419. 17079711 10.1242/jeb.02540

[79] McGraw KJ, Klasing KC (2006) Carotenoids, Immunity, and Integumentary Coloration in Red Junglefowl (*Gallus Gallus*). Auk 123:1161 10.1642/0004-8038(2006)1231161:CIAICI2.0.CO2)

[80] McGraw KJ, Crino OL, Medina-Jerez W, Nolan PM (2006) Effect of Dietary Carotenoid Supplementation on Food Intake and Immune Function in a Songbird with no Carotenoid Coloration. Ethology 112:1209–1216 10.1111/j.1439-0310.2006.01280.x)

[81] Berthouly A, Helfenstein F, Richner H (2007) Cellular immune response, stress resistance and competitiveness in nestling great tits in relation to maternally transmitted carotenoids. Funct Ecol 21:335–343 10.1111/j.1365-2435.2006.01236.x)

[82] Remeš V, Krist M, Bertacche V, Stradi R (2007) Maternal carotenoid supplementation does not affect breeding performance in the Great Tit (*Parus major*). Funct Ecol 21:776–783 10.1111/j.1365-2435.2007.01277.x)

[83] Thorogood R, Kilner RM, Karadaş F, Ewen JG (2008) Spectral mouth colour of nestlings changes with carotenoid availability. Funct Ecol 22:1044–1051 10.1111/j.1365-2435.2008.01455.x)

[84] Ewen JG, Thorogood R, Brekke P, Cassey P, Karadas F, et al (2009) Maternally invested carotenoids compensate costly ectoparasitism in the hihi. Proc Natl Acad Sci U S A 106:12798–802 10.1073/pnas.0902575106) 19620733 PMC2722293

[85] Helfenstein F, Losdat S, Møller AP, Blount JD, Richner H (2010) Sperm of colourful males are better protected against oxidative stress. Ecol Lett 13:213–22 10.1111/j.1461-0248.2009.01419.x) 20059524

[86] Hill EG (1966) Effects of methionine, menhaden oil and ethoxyquin on serum cholesterol of chicks. J Nutr 89:143–148. 5944092 10.1093/jn/89.2.143

[87] Manson MM, Green JA, Wright BJ, Carthew P (1992) Degree of ethoxyquin-induced nephrotoxicity in rat is dependent on age and sex. Arch Toxicol 66:51–6. 1580794 10.1007/BF02307270

[88] Harding CF (2004) Hormonal modulation of singing: hormonal modulation of the songbird brain and singing behavior. Ann NY Acad Sci 1016:524–539 10.1196/annals.1298.030) 15313793

[89] Garamszegi LZ, Eens M (2004) Brain space for a learned task: strong intraspecific evidence for neural correlates of singing behavior in songbirds. Brain Res Rev 44:187–193 10.1016/j.brainresrev.2003.12.001) 15003393

